# GPR43 regulates marginal zone B‐cell responses to foreign and endogenous antigens

**DOI:** 10.1111/imcb.12399

**Published:** 2020-09-28

**Authors:** Leona Rohrbeck, Monika Adori, Shan Wang, Chenfei He, Christopher A Tibbitt, Mark Chernyshev, Madle Sirel, Ulf Ribacke, Ben Murrell, Mohammad Bohlooly‐Y, Mikael CI Karlsson, Gunilla B Karlsson Hedestam, Jonathan M Coquet

**Affiliations:** ^1^ Department of Microbiology, Tumor and Cell Biology (MTC) Karolinska Institutet Stockholm Sweden; ^2^ Translational Genomics, Discovery Sciences BioPharmaceuticals R&D, AstraZeneca Gothenburg Sweden

**Keywords:** Fiber, GPR43, marginal zone B cells, polysaccharide vaccine, short‐chain fatty acids

## Abstract

Marginal zone (MZ) B cells are innate‐like B cells that produce polyreactive antibodies with an affinity for microbial molecular patterns and carbohydrate ligands. MZ B cells have been shown to be important in mediating immunity to various bacteria including *Streptococcus pneumoniae* and are also implicated in inflammatory syndromes including lupus erythematosus. The intestinal microbiota is responsible for producing short‐chain fatty acids, which can regulate immune cell function by several mechanisms including ligation of the G‐protein‐coupled receptor (GPR)43. Herein, we show that MZ B cells express *Gpr43* messenger RNA and that the absence of this receptor impacts on MZ B‐cell surface marker expression and antibody production. In T‐cell‐independent responses to the hapten 4‐hydroxy‐3‐nitrophenylacetic acid (NP), mice deficient in GPR43 displayed higher serum titers of NP‐specific antibodies. Moreover, in response to a pneumococcal polysaccharide vaccine, GPR43‐deficient mice developed robust serum antibody responses and had markedly increased numbers of splenic antibody‐secreting cells, compared with control mice. Finally, serum immunoglobulin M autoantibodies to double‐stranded DNA and phosphatidylcholine were increased in resting 10–15‐week‐old mice lacking GPR43. Taken together, mice lacking GPR43 have heightened antibody responses to T‐cell‐independent antigens, which may be a result of impaired regulation of MZ B cells.

## Graphical abstract

Marginal zone B cells express the short‐chain fatty acid receptor GPR43. Mice lacking GPR43 have heightened responses to T‐cell‐independent antigens and have elevated levels of immunoglobulin M specific for double‐stranded DNA and phosphatidylcholine. Thus, short‐chain fatty acids may regulate marginal zone B‐cell responses to several antigens.

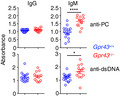

## INTRODUCTION

B cells can be broadly subdivided into three types: B‐1, marginal zone (MZ) B and follicular (Fo) B cells.^1^ Innate B‐1 cells develop from the fetal liver and reside in large frequencies in the pleural and peritoneal cavities where they are self‐renewing. These cells express B‐cell antigen receptors that are enriched for polyreactivity, which afford early protection to several pathogens invading the body such as *Streptococcus pneumoniae* and *Salmonella*. Fo B cells express highly diverse monoreactive B‐cell receptors, and are a major cell type in secondary lymphoid organs.^1^ Fo B cells undergo expansion after stimulation through the B‐cell receptor and following interactions with cognate T cells in germinal centers, undergo rounds of somatic hypermutation leading to isotype‐switched, high‐affinity antibodies against pathogens including bacteria and viruses. Finally, MZ B cells reside in the splenic MZ, adjacent to the red pulp, where they are exposed to blood‐borne infections and particulate matter.^2^, ^3^ Like B‐1 cells, MZ B cells express polyreactive B‐cell receptors and are primarily thought to mediate short‐term immunity to pathogens including *Streptococcus pneumoniae*, *Haemophilus influenzae* and *Neisseria meningitidis*.^2^ Although MZ B cells are typically thought to afford early protection to invading pathogens, MZ B cells can also shuttle antigens into follicles to promote Fo B‐cell responses.^3^, ^4^, ^5^ Furthermore, MZ B cells can produce antibodies that recognize some forms of DNA and phosphoproteins, which can be expressed on the surface of apoptotic cells. The production of antibodies to endogenous particulate matter is proposed to have a beneficial role in homeostasis; however, such antibodies are also postulated to promote pathogenesis in certain inflammatory diseases including lupus and various cardiac disorders.^6^ Thus, finding regulators of MZ B cells may help to define strategies that dampen or prevent autoimmune pathology, while retaining the homeostatic function of natural antibodies produced by these cells.

Short‐chain fatty acids (SCFAs), typically acetate, butyrate and propionate, are produced by saccharolytic bacteria such as Firmicutes and Bacteroidetes.^7^ SCFAs can be generated through a number of sources including fiber‐rich diets and are produced at an approximately 3:1:1 acetate:butyrate:propionate molar ratio in the proximal colon.^7^ Most SCFAs are rapidly absorbed into intestinal epithelial cells after secretion into the gut lumen, although some make it into the bloodstream, with high levels of variability reported within the population.^8^ Preclinical studies where SCFAs or high‐fiber diets are fed to mice have demonstrated that SCFAs may alleviate autoimmune and/or inflammatory pathologies of the bowels, pancreas, lungs and others organs.^9^, ^10^, ^11^ Observational studies of populations with different diets have indicated that people with a high‐fiber diet have increased levels of SCFA,^12^, ^13^ leading to the commencement of several clinical trials to test the efficacy of high‐fiber diets in autoimmune and inflammatory settings such as diabetes, cirrhosis, intestinal inflammatory disorders and cardiovascular disease.

Many effects of SCFAs are related directly to their impact on intestinal epithelium, where they modulate cellular metabolism, gene expression and promote barrier integrity.^14^ However, there is an increased appreciation that SCFAs regulate lymphocyte functions and immunity. Several studies have shown that SCFAs may promote B‐cell responses to intestinal commensals and ingested antigens.^15^, ^16^ In mice fed high‐fiber diets, an increase in immunoglobulin A (IgA)‐producing B cells and decrease in IgE secretion were observed. Furthermore, mice fed SCFAs were shown to develop increased frequencies and numbers of IgA‐secreting plasma cells in the intestinal *lamina propria*, owing to a boost in oxidative and glycolytic metabolism in B cells.^15^, ^16^ However, conflicting reports on the impact of SCFAs on antibody production exist. In two independent studies, butyrate and propionate have been shown to lower *Aicda* (gene for activation‐induced deaminase) expression and reduce plasma cell differentiation.^17^, ^18^ SCFAs were shown to reduce the level of somatic hypermutation and these effects were thought to rely on the induction of microRNAs.^18^ Differences in reports could be a result of a range of factors, including large differences in microbial flora between animal facilities worldwide, which would affect the levels of SCFAs in the gut lumen and in circulation.

Another complicating factor in understanding the role of SCFAs in health relates to their mechanism(s) of action. SCFAs may passively diffuse into cells or be actively transported via the proton‐coupled monocarboxylate transporter 1 and the sodium‐coupled monocarboxylate transporter 1.^14^ Another mode by which they mediate their function is by activating G‐protein‐coupled receptors (GPRs) including GPR41, GPR43 and GPR109a. Activation of SCFA receptors has been shown to have a variety of effects typical of GPRs, including the inhibition of cyclic adenosine monophosphate production and induction of calcium influx into the cytoplasm.^19^ Mechanistically, activation of GPR43 has been shown to promote the differentiation of IgA^+^ plasma cells in the gut, because they are reduced in frequency in GPR43‐deficient mice.^16^ Moreover, SCFA receptors have been shown to promote the functions and persistence of suppressive regulatory T and effector CD8 T cells.^20^, ^21^, ^22^, ^23^

In genome‐wide expression analyses performed as part of the ImmGen consortium,^24^ MZ B cells are proposed to express high levels of *Gpr43* messenger RNA. Thus, we sought to understand the function of this receptor in MZ B‐cell development and responses. Our findings show that MZ B cells develop normally in the absence of GPR43. However, the expression of several cell surface receptors was reduced on MZ B cells, indicative of an altered state. Furthermore, IgM responses to T‐cell‐independent antigens, including 4‐hydroxy‐3‐nitrophenylacetic acid (NP)‐Ficoll and PNEUMOVAX, were significantly enhanced in mice lacking GPR43. Finally, mice lacking GPR43 appeared to have higher circulating autoantibody levels to endogenous antigens including double‐stranded DNA (dsDNA) and phosphatidylcholine (PC). Thus, GPR43 appears to restrain MZ B‐cell responses against a number of antigens, which may have important implications for autoimmune disease.

## RESULTS

### MZ B cells express Gpr43 and GPR43‐deficient mice have elevated basal levels of serum IgM specific for dsDNA and PC

**Figure 1:**
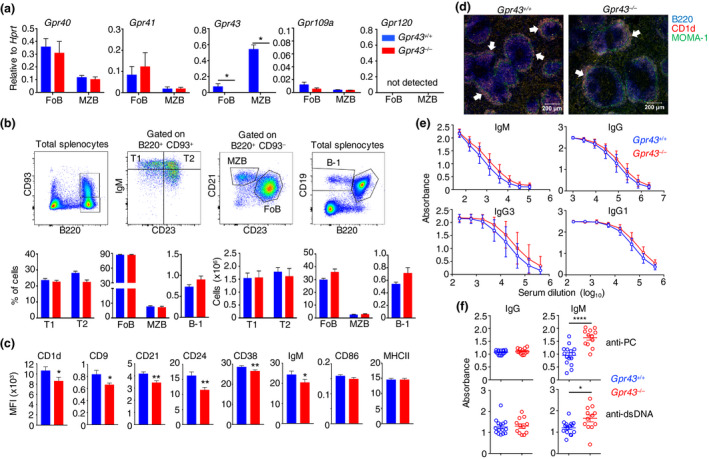
Marginal zone (MZ) B cells express Gpr43 and Gpr43^−/−^ mice have heightened levels of serum immunoglobulin M (IgM) specific to double‐stranded DNA (dsDNA) and phosphatidylcholine (PC). **(a)** Messenger RNA from fluorescence‐activated cell sorting‐purified follicular B (Fo B) or MZ B cells from *Gpr43* ^+/+^ and *Gpr43* ^−/−^ mice was subjected to quantitative reverse transcription‐PCR analysis to measure the expression of *Gpr40, Gpr41, Gpr43*, *Gpr120* and*Gpr109a*. All data are shown as mean values ± s.e.m. of two independent experiments (*n* = 4 mice per group), except for *Gpr109a* which was quantified in a separate experiment with *n* = 4 *Gpr43* ^+/+^ and *n* = 6 *Gpr43* ^−/−^. **(b)** Splenocytes of *Gpr43* ^+/+^ and *Gpr43* ^−/−^ mice were analyzed by flow cytometry. B220^+^CD93^+^ immature or B220^+^CD93^−^ mature B‐cell population are gated. B‐cell precursors within the immature population were further divided into T1 (IgM^+^CD23^−^) or T2 (IgM^+^CD23^+^) cells. The mature B‐cell population was further stained for CD21 and CD23 to divide cells into Fo B (CD21^−^CD23^+^) or MZ B cells (CD21^+^CD23^−^). B‐1 cells were identified as CD19^+^B220^−/low^ cells. Bar graphs show the frequencies or absolute cell numbers of T1, T2, Fo B, MZ B and B‐1 cells. **(c)** Splenocytes from *Gpr43* ^+/+^ and *Gpr43* ^−/−^ mice were stained for MZ B cell markers and mean fluorescence intensity (MFI) levels of the indicated surface molecules are plotted. Data are shown as mean values ± s.e.m. of two independent experiments (*n* = 5 or 6 mice per group). **(d)** Immunofluorescence staining of spleen sections from 10‐ to 12‐week‐old *Gpr43* ^+/+^ and *Gpr43* ^−/−^ mice with B220 (blue) for B cells, CD1d (red) for MZ B cells and MOMA‐1 (green) for marginal metallophilic macrophages. The MZ B‐cell layer is marked by white arrows. Slides are representative of four mice per group. **(e, f)** The serum of 15 *Gpr43* ^+/+^ and 12 *Gpr43* ^−/−^ 10–15‐week‐old naïve mice was screened for the presence of total IgM, IgG, IgG3 and IgG1 at various serum dilutions **(e)** and dsDNA‐ and PC‐specific IgG and IgM at serum concentration of 1:50 **(f)**. The Mann–Whitney *U*‐test was used for comparison, *P < 0.05, **P < 0.01, ****P < 0.0001.

To investigate the expression of SCFA and long‐chain fatty acid receptors, we purified MZ B (B220^+^CD93^−^CD21^+^CD23^−^) and Fo B (B220^+^CD93^−^CD21^−^CD23^+^) cells from the spleens of *Gpr43* ^+/+^ and *Gpr43* ^−/−^ mice.^20^ *Gpr40*, *Gpr41*, *Gpr43*, *Gpr109a* and *Gpr120* messenger RNA was quantified by real‐time PCR. We found that *Gpr43* was expressed at higher levels in MZ B cells compared with Fo B cells and expression of *Gpr43* messenger RNA was completely absent in MZ B cells from *Gpr43* ^−/−^ mice (Figure 1a), confirming the genotype of these mice. Next, we analyzed the spleens of *Gpr43* ^+/+^ and *Gpr43* ^−/−^ mice for the presence and phenotype of MZ B cells by flow cytometry and immunohistochemistry. The numbers of transitional (T)1, T2, Fo B, MZ B and B‐1 cells were similar between *Gpr43* ^+/+^ and *Gpr43* ^−/−^ mice (Figure 1b). B‐1‐cell frequencies also appeared normal in the peritoneal cavity of *Gpr43* ^+/+^ and *Gpr43* ^−/−^ mice (Supplementary figure 1). We noted that the expression of several cell surface receptors expressed by MZ B cells including CD1d, CD9, CD21, CD24, CD38 and IgM was significantly lower in *Gpr43* ^−/−^ compared with control mice (Figure 1c). Analysis of the spleen by immunofluorescence with antibodies to CD1d, B220 and MOMA showed that the structure of the MZs in the spleen appeared normal in *Gpr43* ^−/−^ mice (Figure 1d). We also analyzed the serum of 10–15‐week‐old mice at resting for total IgM, IgG, IgG3 and IgG1 and for the presence of autoantibodies to dsDNA and PC, because these can indicate differences in the function of MZ B cells. While basal serum Ig levels were similar between *Gpr43* ^+/+^ and *Gpr43* ^−/−^ mice (Figure 1e), higher levels of dsDNA‐ and PC‐specific IgM were present in the serum of *Gpr43* ^−/−^ mice compared with control mice (Figure 1f). Thus, the absence of GPR43 does not affect the development of MZ B cells, but may affect their phenotype and function.

### Gene expression and responsiveness to lipopolysaccharide appear normal in MZ B cells from GPR43‐deficient mice

**Figure 2:**
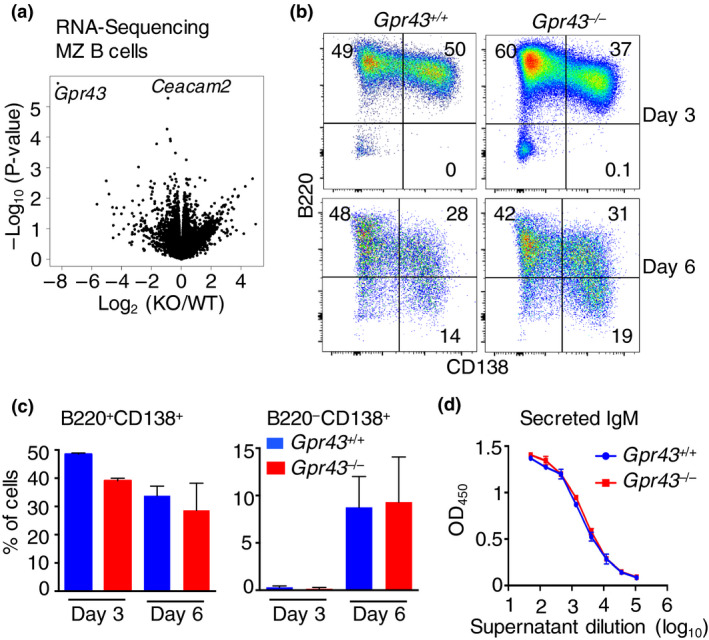
Marginal zone (MZ) B cells from Gpr43^−/−^ mice have normal responsiveness to lipopolysaccharide (LPS). **(a)** MZ B cells from three *Gpr43* ^+/+^ and three *Gpr43* ^−/−^ mice were purified by flow cytometry, messenger RNA was isolated and RNA‐sequencing was performed. A volcano plot depicts the gene expression differences in MZ B cells. Only *Gpr43* and *Ceacam2* were significantly reduced in *Gpr43* ^−/−^ MZ B cells after adjustment for the number of comparisons. **(b–d)** MZ B cells from *Gpr43* ^+/+^ and *Gpr43* ^−/−^ mice were purified and subsequently stimulated with 10 µg mL^−1^ LPS for 3 or 6 days. **(b, c)** Flow cytometry was used to determine differentiation into plasmablasts (B220^+^CD138^+^) and plasma cells (B220^−^CD138^+^). **(d)** Secreted IgM was measured by ELISA. In **b–** **d**, *n* = 3 or 4 mice per group and data are representative of two independent experiments. KO, knockout; OD, optical density; WT, wild type.

RNA‐sequencing on purified splenic MZ B cells from *Gpr43* ^−/−^ and *Gpr43* ^+/+^ mice was conducted to determine whether gene expression was altered in MZ B cells lacking GPR43. Gene transcription profiles of MZ B cells from *Gpr43* ^+/+^ and *Gpr43* ^−/−^ mice were largely overlapping because only *Gpr43* and *Ceacam2* were significantly reduced in the absence of GPR43, after adjustment for multiple comparisons (Figure 2a). We next tested the responsiveness of purified *Gpr43* ^−/−^ MZ B cells to lipopolysaccharide *in vitro*. MZ B cells from *Gpr43* ^+/+^ and *Gpr43* ^−/−^ mice differentiated comparably into plasmablasts (B220^+^CD138^+^) and plasma cells (B220^−^CD138^+^) following culture over 6 days and secreted similar quantities of IgM (Figure 2b–d), suggesting that responsiveness to lipopolysaccharide was normal.

### Enhanced antibody production in *Gpr43* ^−/−^ mice in a T‐cell‐independent immunization model

**Figure 3:**
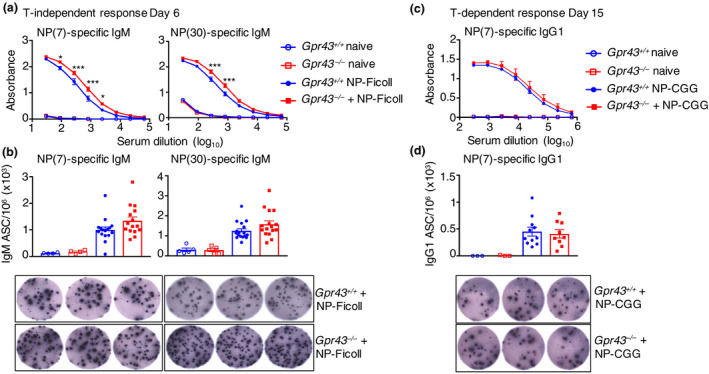
Gpr43^−/−^ mice have heightened responses to T‐cell‐independent antigens while the response to T‐cell‐dependent antigens is unaffected. **(a)** *Gpr43* ^+/+^ and *Gpr43* ^−/−^ mice were immunized intravenously with NP‐Ficoll. Six days after immunization, mice were analyzed for serum immunoglobulin M (IgM) to NP(7) or NP(30) by ELISA. **(b)** NP(7)‐ or NP(30)‐specific IgM antibody‐secreting cells (ASCs) from spleens were measured by ELISpot. Representative wells from splenocytes of *Gpr43* ^+/+^ and *Gpr43* ^−/−^ mice are shown. **(c)** *Gpr43* ^+/+^ and *Gpr43* ^−/−^ mice were injected with NP‐CGG in alum. Seven days later, mice received a boost of NP‐CGG diluted in phosphate‐buffered saline. Fifteen days after the first injection, blood was collected and NP(7)‐specific serum IgG1 levels were measured by ELISA. **(d)** NP(7)‐specific IgG1 ASCs from spleens were measured by ELISpot. Representative wells from splenocytes of *Gpr43* ^+/+^ and *Gpr43* ^−/−^ mice are shown. Three independent experiments were performed with NP‐Ficoll and two independent experiments were performed with NP‐CGG; *n* = 3 or 6 NP‐immunized mice per group per experiment, *n* = 1 or 2 naïve mice per group per experiment as controls. For ELISA, representative data from one experiment are shown and two‐way ANOVA and Šidák’s test were used for comparison. For ELISpot analysis, bar graphs show the pooled results over all experiments and groups were compared using the Mann–Whitney *U*‐test. *P < 0.05, ***P < 0.001. NP, 4‐hydroxy‐3‐nitrophenylacetic acid.

MZ B cells typically make antibodies in the first week of an immune response and do not require activatory signals from T cells. Thus, we analyzed responses to the hapten NP in T‐cell‐independent (NP‐Ficoll) and T‐cell‐dependent [NP‐Chicken Gamma Globulin (CGG)] immunization models. We used high (NP30) and low (NP7) valency NP to detect the quality of the antibody response to NP. At day 6 after immunization with NP‐Ficoll, serum from *Gpr43* ^−/−^ mice contained significantly higher IgM specific to both NP(7) and NP(30), compared with serum from *Gpr43* ^+/+^ mice (Figure 3a). Quantification of IgM‐secreting NP‐specific splenic B cells by ELISpot analysis showed no significant difference between *Gpr43* ^+/+^ and *Gpr43* ^−/−^ mice, although levels were slightly higher in GPR43‐deficient mice (Figure 3b). Analysis of T‐cell‐dependent B‐cell responses following immunization with NP‐CGG in alum demonstrated no clear difference in the quantity of NP(7)‐specific IgG1 present in the serum of *Gpr43* ^+/+^ and *Gpr43* ^−/−^ mice (Figure 3c), nor in the quantity of NP(7)‐specific IgG1‐secreting cells in the spleen (Figure 3d). Thus, GPR43 appeared to restrain MZ B‐cell antibody responses to NP‐Ficoll immunization, but had little effect on Fo B‐cell responses to NP‐CGG.

### Enhanced antibody production in *Gpr43* ^−/−^ mice to pneumococcal antigens

**Figure 4:**
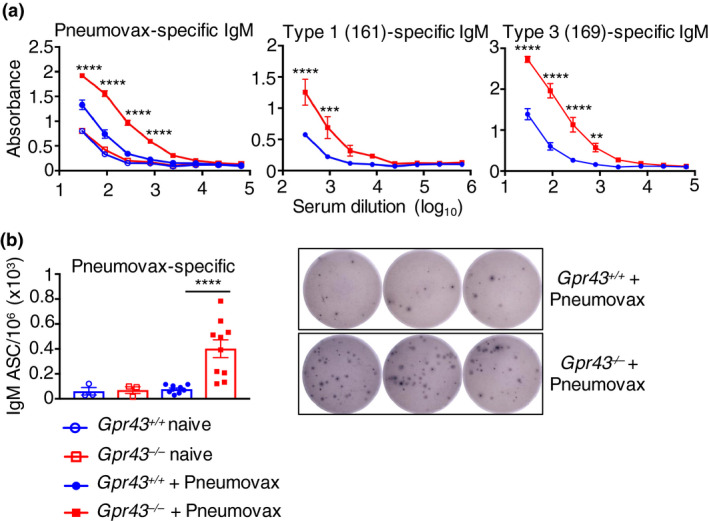
Gpr43^−/−^ mice have heightened responses to immunization with a pneumococcal polysaccharide vaccine. *Gpr43* ^+/+^ and *Gpr43* ^−/−^ mice were immunized intraperitoneally with PNEUMOVAX pneumococcal polysaccharide vaccine and killed after 6 days. The serum immunoglobulin M (IgM) antibody response to **(a)** PNEUMOVAX, type 1 polysaccharide antigen 161‐X or type 3 antigen 169‐X was measured by ELISA. **(b)** The number of PNEUMOVAX‐specific IgM antibody‐secreting cells from the spleen was measured by ELISpot. Representative wells from splenocytes of *Gpr43* ^+/+^ and *Gpr43* ^−/−^ mice are shown. For ELISA, two‐way ANOVA and Šidák’s test were used and in ELISpot, the Mann–Whitney *U*‐test was used to compare groups. Results represent mean values ± s.e.m. of two independent experiments (*n* = 5 PNEUMOVAX‐immunized and 1 or 2 naïve control mice per group per experiment). **P < 0.01, ***P < 0.001, ****P < 0.0001.

We next evaluated the response of *Gpr43* ^−/−^ mice to pneumococcal polysaccharide antigens (PNEUMOVAX). Mice were injected intraperitoneally with PNEUMOVAX and 6 days later serum and spleens were harvested and analyzed for the presence of antibodies and antibody‐secreting cells. PNEUMOVAX‐specific IgM was present in the serum of *Gpr43* ^−/−^ mice in much greater quantities than in *Gpr43* ^+/+^ mice (Figure 4a). Serum antibodies to type 1 (161) and type 3 (169) carbohydrates were also significantly increased in mice lacking GPR43 (Figure 4a), indicative of heightened MZ B‐cell responses in these mice. Analysis by ELISpot also revealed the clear presence of PNEUMOVAX‐specific antibody‐secreting cells in the spleen of *Gpr43* ^−/−^ mice (Figure 4b), whereas antibody‐secreting cells were not detected in appreciable amounts in *Gpr43* ^+/+^ mice.

Taken together, these results suggest that GPR43 restrains MZ B‐cell responses to T‐cell‐independent antigens and reduces antibody responses to endogenous dsDNA and PC.

### Discussion

SCFAs have been shown to have a range of immune modulatory functions, in particular in the gut and associated lymphoid organs. Because SCFAs can effectuate change via various mechanisms, it can be difficult to pinpoint how immune function is modulated. The results herein suggest that SCFAs regulate the function and maturation of MZ B cells in the spleen by activating GPR43. This finding may have important implications in the setting of lupus erythematosus and in various cardiovascular disorders. In both disease settings, antibodies that recognize phospholipids have been suggested to enhance the formation of clots and promote the development of antibody complexes, which can have deleterious effects on the health of several organs including the heart and kidneys. In a recent study of autoimmunity in lupus‐prone mice, addition of SCFAs into drinking water significantly reduced oligonucleotide‐specific antibody titers.^18^ Although the authors suggested that the impact of SCFAs was independent of GPR43, our results support the possibility that SCFAs regulate antibody production to dsDNA, in part via GPR43.

Trials of high‐fiber diets have been started in various clinical settings with the aim of reducing inflammatory symptoms. However, the impact of high‐fiber diets or SCFA supplementation on inflammatory disorders in mice has often yielded conflicting results.^25^ This may be because of the broad mechanisms of action of SCFAs. Agonists and antagonists of SCFA receptors have been characterized over recent years, which may provide a more targeted therapeutic option going forward.^26^ For instance, mice treated with a GPR43 agonist were shown to be more resistant to dextran sulfate sodium‐induced colitis.^23^ Furthermore, GPR43 agonists have been shown to boost insulin secretion from pancreatic beta cells^27^ and at the same time, inhibit insulin signaling into adipocytes, thereby preventing lipid accumulation in adipose tissue.^28^ Whether GPR43 (or other SCFA receptors) is expressed by human MZ B cells, and whether they play a regulatory role, is yet to be explored.

Although our study implies that GPR43 may directly regulate MZ B‐cell antibody responses, it is possible that B‐1 cells, which also produce antibodies to carbohydrate antigens and to circulating dsDNA and PC,^29^ may be altered in function in mice lacking GPR43. Furthermore, GPR43 may regulate MZ B‐cell functions at various levels. For instance, whether GPR43 regulates B‐cell receptor signaling into MZ B cells remains unclear. Reduced expression of receptors including CD1d and CD21 on MZ B cells also allude to the possibility that other MZ B‐cell functions are altered in the absence of GPR43, including antigen presentation to natural killer cells^30^ and potentially, antigen capture and transport into lymph node follicles.^4^, ^5^ The impact of GPR43 on these functions should be investigated. In all, this study suggests that GPR43 restricts antibody responses to T‐cell‐independent antigens and dampens antibody responses to the autoantigens dsDNA and PC.

## METHODS

### Mice

C57BL/6N wild‐type mice (*Gpr43* ^+/+^) were purchased from Janvier Labs (Rennes, France). *GPR43*‐deficient mice (*Gpr43* ^−/−)^ were sourced from Astra Zeneca. Mice were housed and bred at Karolinska Institutet animal facility. All animal procedures were approved by the Stockholms Jordbruksverket in Sweden and performed according to approved guidelines.

### Flow cytometry and cell sorting

Single‐cell suspensions from indicated tissues or cell cultures were washed in phosphate‐buffered saline (PBS)–1% fetal bovine serum. Cells were subsequently incubated with anti‐CD16/32 Ab (2.4G2), followed by staining with biotin‐ or fluorochrome‐conjugated antimouse antibodies from either BD Biosciences (Franklin Lakes, NJ, USA) or BioLegend (San Diego, CA, USA) diluted in cold PBS plus 1% fetal calf serum: B220 (RA3‐6B2), CD93 (AA4.1), CD21 (7E9), CD23 (B3B4), CD138 (281‐2), CD4 (RM4‐5), CD5 (53‐7.3), CD24 (M1/69), CD1d (1B1), CD9 (KMC8), CD19 (1D3), CD43 (S7), IgD (11‐26c.2a), IgM (RMM‐1), CD38 (70) and CD86 (GL1). Biotinylated antibodies were detected using streptavidin–Alexa Fluor 488 (Invitrogen, Eugene, OR, USA) or streptavidin–Percp‐Cy5.5 (BD Biosciences). Cells were analyzed using the LSR II (BD Biosciences) flow cytometer or sorted on the FACSAria Fusion (BD Biosciences), and data were analyzed using the FlowJo software version 10.0.8 (Tree Star, Ashland, OR, USA).

### Quantitative reverse transcription‐PCR

Cell pellets were resuspended in TRIzol reagent (Life Technologies, Carlsbad, CA, USA) and RNA was isolated following the manufacture’s protocol. RNA was reverse transcribed into complementary DNA utilizing the Super Script IV First Strand Complementary DNA Synthesis kit (Thermo Fisher Scientific, Waltham, MA, USA). Quantification of *Ffar1 (Gpr40), Ffar2 (Gpr43), Ffar3 (Gpr41), Ffar4 (Gpr120), Gpr109a* or *Hprt* was performed using SYBR Green dye‐based real‐time PCR and samples were run on a CFX384 machine (Bio‐Rad, Hercules, CA, USA). Data were analyzed using the 2ΔΔCt method where *Hprt* was used as a housekeeping gene.

### Immunization with NP‐Ficoll, PNEUMOVAX or NP‐CGG

Age and sex‐matched *Gpr43* ^+/+^ and *Gpr43* ^−/−^ mice were injected intravenously with 50 µg of 2,4,6‐trinitrophenyl‐Ficoll (TNP‐AECM‐FICOLL; Biosearch Technologies, Novato, CA, USA), intraperitoneally with NP‐CGG in alum or intraperitoneally with a 1:10 dilution of PNEUMOVAX (MSD, Merck Sharp and Dohme Corp, Kenilworth, NJ, USA), which corresponds to 0.5 µg of each polysaccharide antigen. The antigens were diluted in sterile PBS and mice were injected with 100 µL. For T‐cell‐independent immunizations, mice were killed 6 days later and spleens and blood were taken for fluorescence‐activated cell sorting analysis, ELISpot and ELISA assays. For T‐cell‐dependent immunization with NP‐CGG, mice were boosted at day 7 and killed at day 15.

### Enzyme‐linked immunosorbent assay

To measure antigen‐specific antibodies, ELISA plates (Nunc) were coated with 5 µg mL^−1^ of NP(7) conjugated with bovine serum albumin (BSA), NP(30) conjugated with BSA, 1:100 dilution of PNEUMOVAX, pneumococcal polysaccharide antigen Type 1 161‐X or Type 3 169‐X, 50 µg mL^−1^ dsDNA (Sigma D‐4522, Merck Sharp and Dohme Corp) with 5 µg mL^−1^ methylated BSA (Sigma A‐1009, Merck Sharp and Dohme Corp) or 2.5 µg mL^−1^ PC‐BSA (Athera Biotechnologies, Stockholm, Sweden). Plates were washed with washing buffer (PBS + 0.05% Tween‐20) and subsequently blocked with PBS containing 5% dry milk at room temperature (RT) for 1 h. Serum or supernatant was diluted in PBS containing 1% dry milk, was added to the plate in threefold serial dilutions and plates were incubated at RT for 1.5 h. After washing six times with washing buffer, 100 µL of diluted secondary horseradish peroxidase‐ or biotin‐coupled goat antimouse IgG, IgG3, IgG1 or IgM antibody (Southern Biotechnology, Birmingham, AL, USA) was added per well and plates were incubated at RT for 1.5 h. For reactions using a biotinylated detection antibody, wells were washed six times and 100 µL of diluted streptavidin–horseradish peroxidase was added to plates and incubated at RT for 1 h. The assay was developed with 3′,5,5′‐Tetramethylbenzidine substrate (KPL, Gaithersburg, MD, USA) and the reaction stopped by adding 1 m H_2_SO_4_. Absorbance was read at 450 nm using an Asys Expert 96 ELISA reader (Biochrom, Cambridge, UK).

### ELISpot

An ELISpot assay was used to detect NP‐ or PNEUMOVAX‐specific IgM‐ or IgG‐producing cells. MultiScreen Filter plates were pretreated with 70% EtOH, washed in sterile PBS and coated with 5 µg mL^−1^ [NP(7)‐BSA or NP(30)‐BSA] or 1:100 PNEUMOVAX diluted in PBS. Plates were incubated overnight at 4°C. The following day, plates were washed in sterile PBS and blocked in complete Roswell Park Memorial Institute medium for 2 h at 37°C. Single‐cell suspensions were prepared and added to the plates in triplicate in twofold dilutions, starting at 5 × 10^5^ cells/well in 100 µL total volume. Plates were wrapped in plastic foil and incubated for 16 h at 37°C in 5% CO_2_. The next day, cells were removed by washing the plates with PBS + 0.05% Tween‐20, followed by incubation with biotinylated goat antimouse IgM or IgG (Mabtech, Nacka Strand, Sweden) antibody diluted in PBS for 2 h at RT. Plates were washed in PBS and ALP‐conjugated streptavidin (Mabtech) diluted in PBS was added followed by incubation for 45 min at RT. After washing plates with water, they were developed with 100 µL filtered 5‐bromo‐4‐chloro‐3‐indolyl phosphate/NBT‐plus substrate (Mabtech). The reaction was stopped by washing plates extensively with tap water. Plates were leftover night to dry and spots were counted using an ELISPOT reader and analyzed using the BioSpot suite (Cellular Technology Limited, Shaker Heights, OH, USA).

### *In vitro* stimulation

About 2 × 10^5^ fluorescence‐activated cell sorting‐sorted MZ B cells or 5 × 10^5^ fluorescence‐activated cell sorting‐sorted FO B cells were cultured in a 48‐well plate (TPP, Trasadingen, Switzerland) in 600 µL Roswell Park Memorial Institute complete medium in the presence of absence of 10 µg mL^−1^ *Escherichia coli* lipopolysaccharide (0222B4; Sigma, Merck Sharp and Dohme Corp). Cells were kept at 37°C in a 5% CO_2_ humidified incubator. On days 3 and 6 of stimulation, cells were harvested for fluorescence‐activated cell sorting analysis and supernatant was taken to measure the IgM titer by ELISA.

### Immunofluorescence

Spleens were harvested, embedded in optimal cutting temperature compound (Bio‐Optica, Milano, Italy), snap frozen and stored at −80°C. Sections (8 µm) were cut using Thermo Scientific CryoStar NX70 Cryostat (Thermo Scientific, Waltham, MA, USA), thaw‐mounted on SuperFrost plus adhesion slides (Thermo Scientific), air‐dried and stored at −80°C until use. Prior to staining, slides were fixed in ice‐cold 100% acetone (Sigma‐Aldrich, Merck Sharp and Dohme Corp) for 5 min. Slides were then blocked with 5% goat serum (Agilent, Santa Clara, CA, USA) in PBS for 30 min. MZs were visualized by staining with anti‐B220‐Pacific Blue (103227, BD Biosciences), anti‐MOMA‐FITC (MCA947F; Bio Rad antibodies, Hercules, CA, USA) and anti‐mouse CD1d antigen PE (123510; BioLegend) overnight at 4°C. Washed slides were mounted with ProLong Diamond Antifade Mountant (Catalog NO. P36961; Invitrogen, Carlsbad, CA, USA). Visual data were acquired with a confocal microscope (LSM880; Zeiss, Oberkochen, Germany) and recorded with the LSM Image software. Images were analyzed with Photoshop.

### RNA‐sequencing and analysis

Sequencing libraries were constructed using the TruSeq Library Prep kit version 2 (Illumina, San Diego, CA, USA) from messenger RNA from purified MZ B cells from *Gpr43* ^−/−^ (*n* = 3) and *Gpr43* ^+/+^ (*n* = 3) mice. Indexed sequence libraries were pooled for multiplexing. Sequencing was performed on an Illumina NextSeq 550 platform as 75‐bp long single‐reads. Running FastQC (https://www.bioinformatics.babraham.ac.uk/projects/fastqc/) on the raw fastq files revealed the presence of adapters, poly‐A tails and poly‐G strings indicative of falsely confident calls on no signal rounds. Trim Galore’s (http://www.bioinformatics.babraham.ac.uk/projects/trim_galore/) auto‐detect adapter option was used to trim adapters, while –a and –a G{10} options were used to trim off poly‐As and poly‐Gs. Trimmed files were run through FastQC and multiQC to verify quality. Alignment program STAR was utilized to map reads onto the GRCm38 genome assembly and RSEM was utilized to calculate expression values. The expression values were read into R, where low‐count genes (less than 1 CPM for 2 or more samples) were eliminated and intersample CPM values were normalized using the method of trimmed mean of M‐values. Differential expression was determined using R’s limma package. After adjustment for multiple comparisons, *Gpr43* (adjusted *P* = 0.023) and *Ceacam2* (adjusted *P* = 0.036) were the only two genes differentially expressed between the MZ B from *Gpr43* ^−/−^ and *Gpr43* ^+/+^ mice.

### Statistical analysis

When comparing two groups, the Mann–Whitney *U‐*test was used. For ELISA results, two‐way ANOVA and Šidák’s test were used to determine statistical differences at different titrations of serum.

## AUTHOR CONTRIBUTION

**Leona Rohrbeck:** Conceptualization; Data curation; Formal analysis; Funding acquisition; Writing‐original draft. **Monika Adori:** Conceptualization; Data curation; Formal analysis; Investigation; Methodology. **Shan Wang:** Data curation; Formal analysis; Investigation. **Chenfei He:** Data curation; Formal analysis; Investigation. **Christopher A Tibbitt:** Investigation; Methodology. **Mark Chernyshev:** Data curation; Formal analysis; Visualization. **Madle Sirel:** Formal analysis; Investigation. **Ulf Ribacke:** Conceptualization; Formal analysis. **Ben Murrell:** Data curation; Formal analysis; Supervision. **Mohammad Bohlooly‐Y:** Resources. **Mikael CI Karlsson:** Methodology; Resources; Supervision; Writing‐original draft. **Gunilla B Karlsson Hedestam:** Conceptualization; Resources; Supervision. **Jonathan M Coquet:** Conceptualization; Resources; Supervision; Writing‐original draft; Writing‐review & editing.

## CONFLICT OF INTEREST

The authors declare no conflict of interest.

## Supporting information

Figure S1
