# Supplementary material for: GPR43 regulates marginal zone B‐cell responses to foreign and endogenous antigens
Source: Immunol Cell Biol. 2020 Sep 28;99(2):234–43. doi: 10.1111/imcb.12399 (PMC7891568; doi:10.1111/imcb.12399)
Supplement: Supplementary file 1 — Figure S1 [file IMCB-99-234-s001.pdf]

Rohrbeck et al.,

Supplementary Data

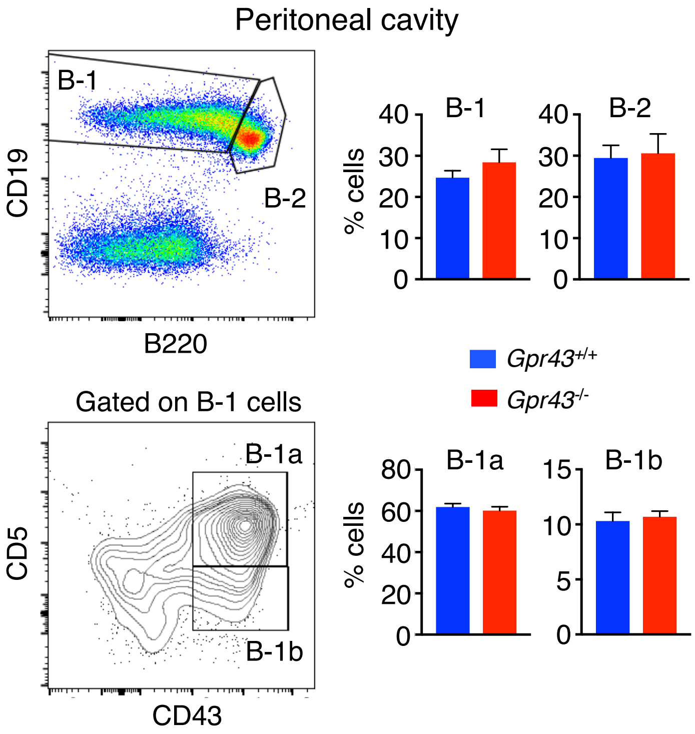

Supplementary Figure 1. Peritoneal cavity B-1 cells are present in normal frequencies in *Gpr43*$^{-/-}$ mice. Peritoneal lavage was performed with 5 ml of PBS and lymphocytes were analysed for the presence of B-1 and B-2 cells using CD19, B220, CD5 and CD43.
